# Discs-large (DLG) is clustered by presynaptic innervation and regulates postsynaptic glutamate receptor subunit composition in *Drosophila*

**DOI:** 10.1186/1741-7007-3-1

**Published:** 2005-01-08

**Authors:** Kaiyun Chen, David E Featherstone

**Affiliations:** 1 Department of Biological Sciences, University of Illinois at Chicago, Chicago, IL 60607, USA

## Abstract

**Background:**

*Drosophila *discs-large (DLG) is the sole representative of a large class of mammalian MAGUKs, including human DLG, SAP 97, SAP102, and PSD-95. MAGUKs are thought to be critical for postsynaptic assembly at glutamatergic synapses. However, glutamate receptor cluster formation has never been examined in *Drosophila *DLG mutants. The fly neuromuscular junction (NMJ) is a genetically-malleable model glutamatergic synapse widely used to address questions regarding the molecular mechanisms of synapse formation and growth. Here, we use immunohistochemistry, confocal microscopy, and electrophysiology to examine whether fly NMJ glutamate receptor clusters form normally in DLG mutants. We also address the question of how DLG itself is localized to the synapse by testing whether presynaptic innervation is required for postsynaptic DLG clustering, and whether DLG localization requires the presence of postsynaptic glutamate receptors.

**Results:**

There are thought to be two classes of glutamate receptors in the *Drosophila *NMJ: 1) receptors that contain the subunit GluRIIA, and 2) receptors that contain the subunit GluRIIB. In DLG mutants, antibody staining for the glutamate receptor subunit GluRIIA is normal, but antibody staining for the glutamate receptor subunit GluRIIB is significantly reduced. Electrophysiological analysis shows an overall loss of functional postsynaptic glutamate receptors, along with changes in receptor biophysical properties that are consistent with a selective loss of GluRIIB from the synapse. In uninnervated postsynaptic muscles, neither glutamate receptors nor DLG cluster at synapses. DLG clusters normally in the complete absence of glutamate receptors.

**Conclusions:**

Our results suggest that DLG controls glutamate receptor subunit composition by selectively stabilizing GluRIIB-containing receptors at the synapse. We also show that DLG, like glutamate receptors, is localized only after the presynaptic neuron contacts the postsynaptic cell. We hypothesize that glutamate receptors and DLG cluster in response to parallel signals from the presynaptic neuron, after which DLG regulates subunit composition by stabilizing (probably indirectly) receptors that contain the GluRIIB subunit. The mechanism(s) stabilizing GluRIIA-containing receptors remains unknown.

## Background

The molecular mechanisms that target postsynaptic glutamate receptors to the postsynaptic membrane, and keep receptors clustered there, remain unknown. Membrane-associated guanylate kinase proteins (MAGUKs) are cell-cell junction proteins with multiple protein-interaction domains (PDZ, SH3, 4.1/Hook, and a catalytically inactive guanylate kinase/GUK domain) [1-3]. Synaptic MAGUKs are widely believed to be required for recruitment and/or stabilization of a variety of synaptic proteins, including glutamate receptors in the postsynaptic density (PSD) [2,4-6]. Although genetic evidence for MAGUK-dependent clustering of NMDA receptors is strongest, and consistent with a model wherein MAGUKs traffick NMDARs to the membrane [7,8], the evidence for scaffolding or trafficking of non-NMDA ionotropic glutamate receptors by MAGUKs is largely based on biochemical interactions and overexpression [9-12]. There is little evidence showing that glutamate receptors fail to cluster appropriately in the absence of MAGUKs – a critical prediction of the 'MAGUK scaffold' model.

*Drosophila *DLG is a prototypical MAGUK, containing three PDZ domains, an SH3 domain, a hook/4.1-binding domain, and a GUK domain [3,13]. DLG is the sole fly representative of a large group of mammalian MAGUKs, including SAP-90/PSD-95, SAP-102/NE-dlg, Chapsyn-110/PSD-93, and SAP97/human DLG [3]. DLG was originally isolated as a tumor suppressor due to loss of apicobasal polarity in *dlg *mutants and consequent tumorous overgrowth in imaginal disc epithelia [14,15]. Since then, DLG has been shown to be present at several types of cell junction, including the glutamatergic larval neuromuscular junction (NMJ) [16-19].

The *Drosophila *NMJ is a widely-used model glutamatergic synapse that is molecularly and developmentally similar to glutamatergic synapses in the mammalian CNS. *Drosophila *NMJs in DLG mutants show a variety of changes, including disrupted organization of synaptic shaker potassium channels and fasciclin II, plus subtle alterations in larval synaptic growth [17,20-22]. It is clear from previous studies that DLG is not absolutely required for glutamate receptor expression and localization in the NMJ. In fact, DLG mutant larvae display larger excitatory postsynaptic potential amplitudes [17]. However, this phenotype depends specifically on presynaptic, but not postsynaptic loss of DLG [17]; presynaptic loss of DLG has subsequently been shown to increase synaptic vesicle diameter and quantal size [23]. Thus, based on measures of NMJ transmission, it is difficult to determine,, whether subtle changes in glutamate receptor cluster formation have occurred. Another complication is that DLG mutant larvae show dramatic underdevelopment of the subsynaptic reticulum (SSR), a dense infolding of postsynaptic membrane that appears during larval NMJ growth [16,17,19,24]. This loss of postsynaptic membrane in DLG mutant larvae makes it difficult to evaluate changes in postsynaptic transmembrane proteins, such as receptors.

Thus, there has so far been no answer to the question of whether DLG is involved in the formation of postsynaptic glutamate receptor clusters in *Drosophila*. However, the aforementioned phenotypic and technical obstructions can be completely avoided in two ways. First, we can examine glutamate receptors in DLG mutant embryos rather than larvae. In embryos, the SSR has not yet formed [24]; therefore there are not yet any differences in postsynaptic membrane abundance between DLG mutant and control NMJs. Second, we can assay postsynaptic glutamate receptors directly, by immunohistochemistry and pressure ejection of glutamate onto voltage-clamped postsynaptic muscle cells [25]. This circumvents any presynaptic alterations. Immunocytochemical techniques are particularly valuable, because antibodies that recognize different receptor subunits can show whether DLG differentially regulates subpopulations of receptors that differ in subunit composition. Mammalian studies have made it increasingly apparent that many aspects of receptor assembly and trafficking depend on the presence of specific subunits. Evidence for molecularly distinct subpopulations of glutamate receptors in *Drosophila *NMJs has only recently been presented [26,27]. Differential regulation of these receptors has never before been demonstrated.

The *Drosophila *NMJ contains five different ionotropic glutamate receptor subunits, each encoded by a different gene: GluRIIA, GluRIIB, GluRIIC (also referred to as 'GluRIII'), GluRIID, and GluRIIE [26-30]. By sequence, fly NMJ subunits are most similar to mammalian kainate receptors. Mutations in GluRIIC, GluRIID, or GluRIIE are lethal, show loss of functional NMJ glutamate receptors, and eliminate immunoreactivity for other subunits [26,27,30]. Thus, GluRIIC, GluRIID, and GluRIIE are thought to be essential subunits contained by each glutamate receptor at the NMJ. In contrast, null mutations in either GluRIIA or GluRIIB individually are viable, but deletion of both GluRIIA and GluRIIB simultaneously is lethal [29,31]. Evidence from ligand binding studies and partial crystal structures strongly suggests that ionotropic glutamate receptors are tetramers [32-34]. Thus, it is thought that *Drosophila *NMJ glutamate receptors are heterotetramers composed of one GluRIIC subunit, one GluRIID subunit, and one GluRIIE subunit, plus either one subunit of GluRIIA or one subunit of GluRIIB [26,27]. This model is consistent with immunocytochemical results: immunoreactivity for GluRIIA only partially overlaps that of GluRIIB [30], suggesting that at least some receptors contain either GluRIIA or GluRIIB, but not both. In other words, the *Drosophila *NMJ contains two subclasses of ionotropic glutamate receptor: 1) GluRIIA-containing receptors and 2) GluRIIB-containing receptors.

Here, we use electrophysiology and immunocytochemistry to demonstrate selective loss of GluRIIB, but not GluRIIA, in DLG mutant *Drosophila *embryos. This is the first demonstration that DLG regulates synaptic glutamate receptor abundance in *Drosophila*, and the first evidence that fly NMJ receptors can be differentially regulated, based on subunit composition. We also explored the mechanisms by which DLG itself is localized at the NMJ. Neither GluRIIA nor GluRIIB are localized unless a presynaptic neuron first contacts the postsynaptic cell. DLG is also not clustered in the absence of presynaptic innervation. This neuronal contact-dependent clustering of DLG does not depend on the clustering or expression of glutamate receptors, because DLG is clustered properly in the absence of all postsynaptic glutamate receptors. Our results are consistent with a model in which an unknown signal from the presynaptic neuron triggers parallel clustering of both DLG and glutamate receptors, after which DLG promotes the synaptic stability of receptors containing GluRIIB, but not GluRIIA.

## Results

### DLG, GluRIIA, and GluRIIB are localized postsynaptically at the *Drosophila *NMJ

**Figure 1:**
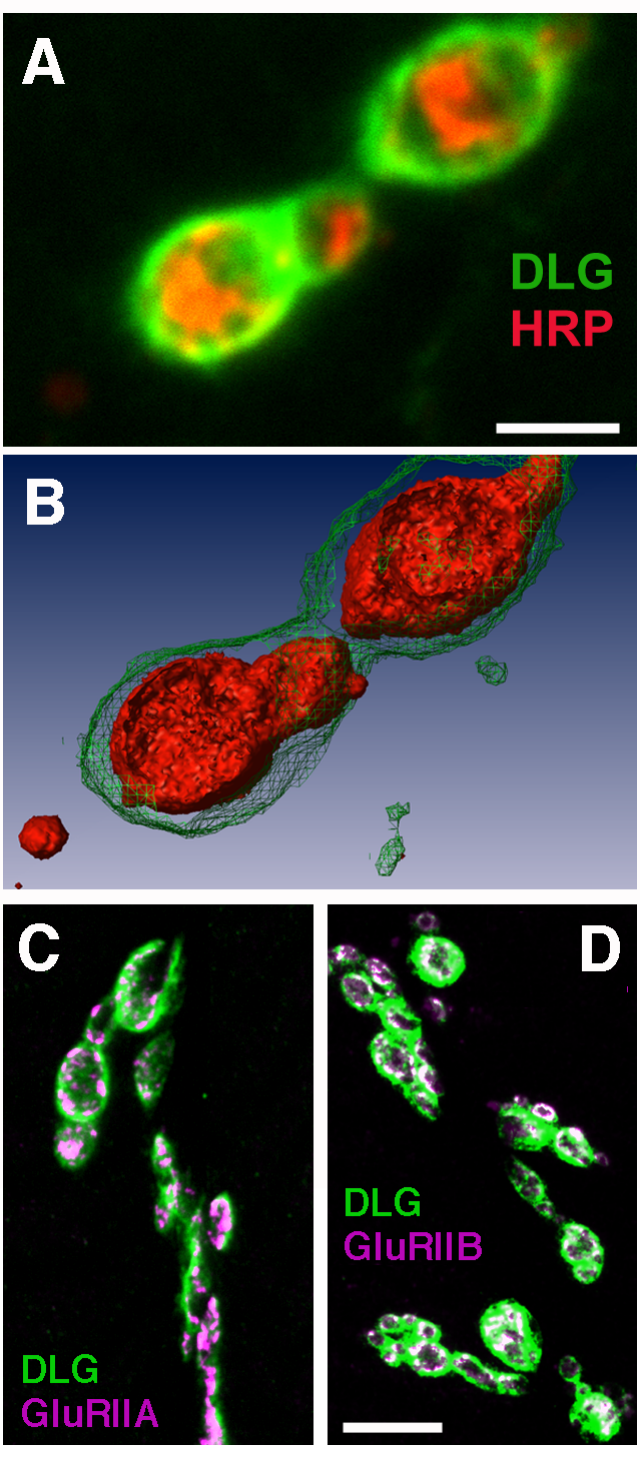
**DLG, GluRIIA, and GluRIIB are localized postsynaptically at the *Drosophila *NMJ **A: Confocal projection of two boutons in a *Drosophila *third instar neuromuscular junction, visualized using the neuronal membrane marker anti-HRP (red) and anti-DLG antibodies (green). Scale bar = 2 μm. B: Isosurface projection generated from the confocal stack shown projected in A. At this stage of development, larval boutons are partially embedded in postsynaptic muscle membrane. DLG immunoreactivity surrounds the boutons, consistent with postsynaptic localization. C, D: Confocal projections of larval NMJs visualized using antibodies that recognize DLG (green) and the glutamate receptor subunits GluRIIA or GluRIIB (magenta, in panels C and D, respectively). Note the incomplete overlap of DLG and glutamate receptors; glutamate receptor immunoreactivity falls within the area stained by DLG, but not all DLG immunoreactivity overlaps with glutamate receptor immunoreactivity. Scale bar = 10 μm.

As previously demonstrated [16-19], DLG is abundantly expressed throughout the postsynaptic membrane surrounding presynaptic motor axon terminals (aka 'boutons') (Fig. 1A–B). DLG appears distributed throughout the postsynaptic membrane; there are no discernible DLG-positive domains smaller than the size of a bouton. In contrast, immunoreactivity for NMJ glutamate receptors has been shown to be restricted to specific postsynaptic domains directly opposite presynaptic active zones [26,35]. This restricted immunoreactivity is visible as distinct clusters within the bouton-wide area delimited by DLG staining (Fig. 1C–D). Thus, not all postsynaptic DLG appears associated with glutamate receptors. We cannot determine by light microscopy whether all glutamate receptors colocalize with DLG, although our staining is consistent with that conclusion.

### GluRIIB, but not GluRIIA, is lost from synapses in DLG mutants

**Figure 2:**
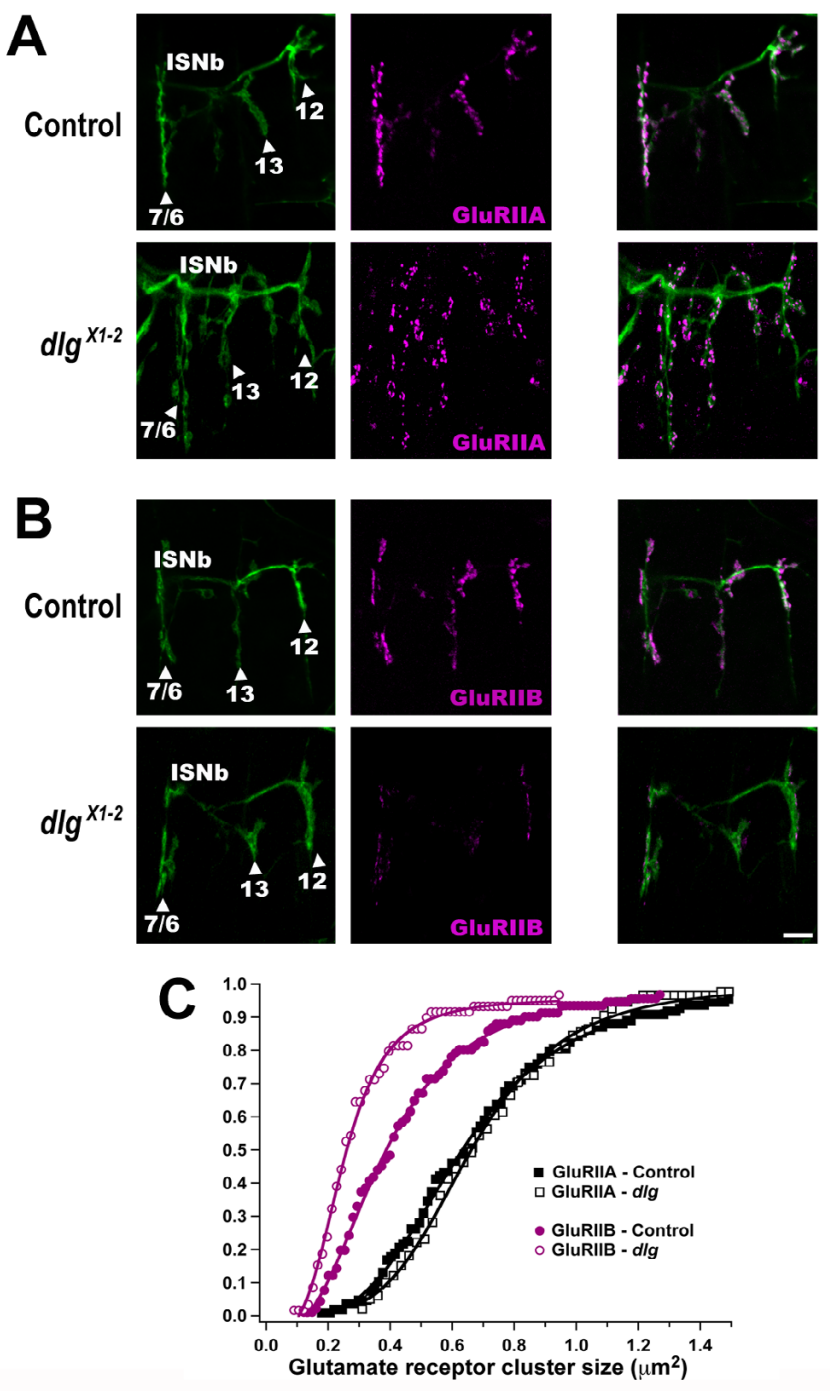
**GluRIIB, but not GluRIIA, is lost from synapses in DLG mutants **A: Confocal projections of late stage 17 embryonic NMJs visualized using antibodies to the neuronal membrane marker anti-HRP (green) and anti-GluRIIA subunit antibodies (magenta). Each panel shows NMJs on interior-most ventral longitudinal muscles in one hemisegment. Major anatomical landmarks are labelled: Intersegmental nerve branch b (ISNb) enters from the left (medial) and branches to form NMJs on muscles 7 & 6, 13, and 12. The top row of panels shows images from control embryos; the lower row of panels shows images from *dlg *mutant embryos. B: As in A, except anti-GluRIIB subunit antibodies (magenta) were used. Scale bar = 5 μm. C: Cumulative frequency plot of glutamate receptor cluster sizes, measured from images such as those shown in A &; B. GluRIIA cluster sizes (black squares) do not differ between control (filled squares) and *dlg *mutant (open squares) embryos. GluRIIB cluster sizes (magenta circles), however, are significantly smaller in *dlg *mutant embryos (open circles), compared to controls (filled circles).

In *Drosophila *embryos and larvae, the intersegmental nerve branch b (ISNb) innervates the ventral longitudinal muscles of each abdominal hemisegment [36]. In the confocal images shown in Fig. 2, ISNb is visualized using anti-HRP antibodies, which stain all neuronal membranes (green). Three NMJs on four muscles (not stained) are shown in each image (Fig. 2A–B). Ventral longitudinal muscles 7 and 6 are innervated via a NMJ that lies in the cleft between the two adjacent muscles. Muscles 13 and 12 are innervated by arborizations distal to the 7/6 NMJ. Each of these body wall NMJs contains multiple clusters of postsynaptic glutamate receptors that can be visualized using antibodies specific to either GluRIIA (Fig. 2A) or GluRIIB (Fig. 2B).

To determine whether DLG is required for clustering of postsynaptic glutamate receptors, we visualized NMJ glutamate receptors in control and DLG mutant embryonic NMJs using GluRIIA and GluRIIB specific antibodies [30]. To manipulate DLG levels, we used embryos homozygous for the mutation *dlg*^*X*1–2^. In *dlg*^*X*1–2 ^mutants, the S97N isoform of DLG, which is predominantly expressed in neurons and muscle, is reduced to undetectable levels [37]. Other isoforms of *dlg *(*Drosophila *expresses at least five) are expressed only at extremely low levels (approximately 5% normal) [37]. In addition, all isoforms (including S97) are truncated such that the C-terminus and GUK domains are completely removed [20].

Both control and *dlg*^*X*1–2 ^mutant NMJs contain highly visible clusters of GluRIIA-containing receptors (Fig 2A; magenta). In *Drosophila *embryonic NMJs, the cluster area is directly proportional to the number of functional postsynaptic receptors measured using patch-clamp electrophysiology [25]. GluRIIA cluster area does not differ significantly between control and *dlg*^*X*1–2 ^mutants (control cluster area = 0.68 ± 0.03 μm^2^, N = 103 clusters from 10 embryos; *dlg *= 0.75 ± 0.03 μm^2^, N = 99 clusters from 16 embryos; P = 0.15). Immunoreactivity for GluRIIB, on the other hand, appears dramatically decreased in *dlg*^*X*1–2 ^mutants compared to controls (Fig. 2B). Indeed, GluRIIB cluster size is significantly decreased in *dlg*^*X*1–2 ^mutants compared to controls (control cluster area = 0.45 ± 0.03 μm^2^, N = 88 clusters from 6 embryos; *dlg *= 0.31 ± 0.02 μm^2^, N = 57 clusters from 6 embryos; P < 0.001). Cumulative frequency histograms of GluRIIA and GluRIIB cluster sizes (Fig. 2C) represent the entire distribution of cluster sizes measured in control and mutant embryos. Fig. 2C shows that the distribution of GluRIIA cluster sizes is almost identical in control and *dlg *mutants. The GluRIIB cluster size curve in *dlg *mutants, however, is shifted toward smaller values. The largest shift occurs in the section of the curve where cluster sizes are largest, suggesting that the largest GluRIIB clusters are preferentially lost in *dlg *mutants. However, the smallest clusters approach the resolution limit of light microscopy, where reductions in object size are no longer detectable. Thus, the reduction in small cluster size is probably underestimated, and the average decrease of GluRIIB in *dlg *mutants may be larger than is measurable by immunocytochemistry.

### Postsynaptic glutamate receptor current properties change in DLG mutants

The immunocytochemistry in Fig. 2 suggests that *dlg*^*X*1–2 ^mutants specifically lose receptors that contain GluRIIB, but do not lose receptors containing GluRIIA. GluRIIA null mutants are viable, but mEJP amplitudes are smaller, glutamate receptor channel open times are reduced, and receptors show decreased sensitivity to the GluR antagonist argiotoxin 636 [31]. GluRIIB null mutants are also viable, but show no significant change in receptor function, suggesting that either the GluRIIB subunit plays a lesser role in channel function, or that the majority of native receptors lack GluRIIB.

**Figure 3:**
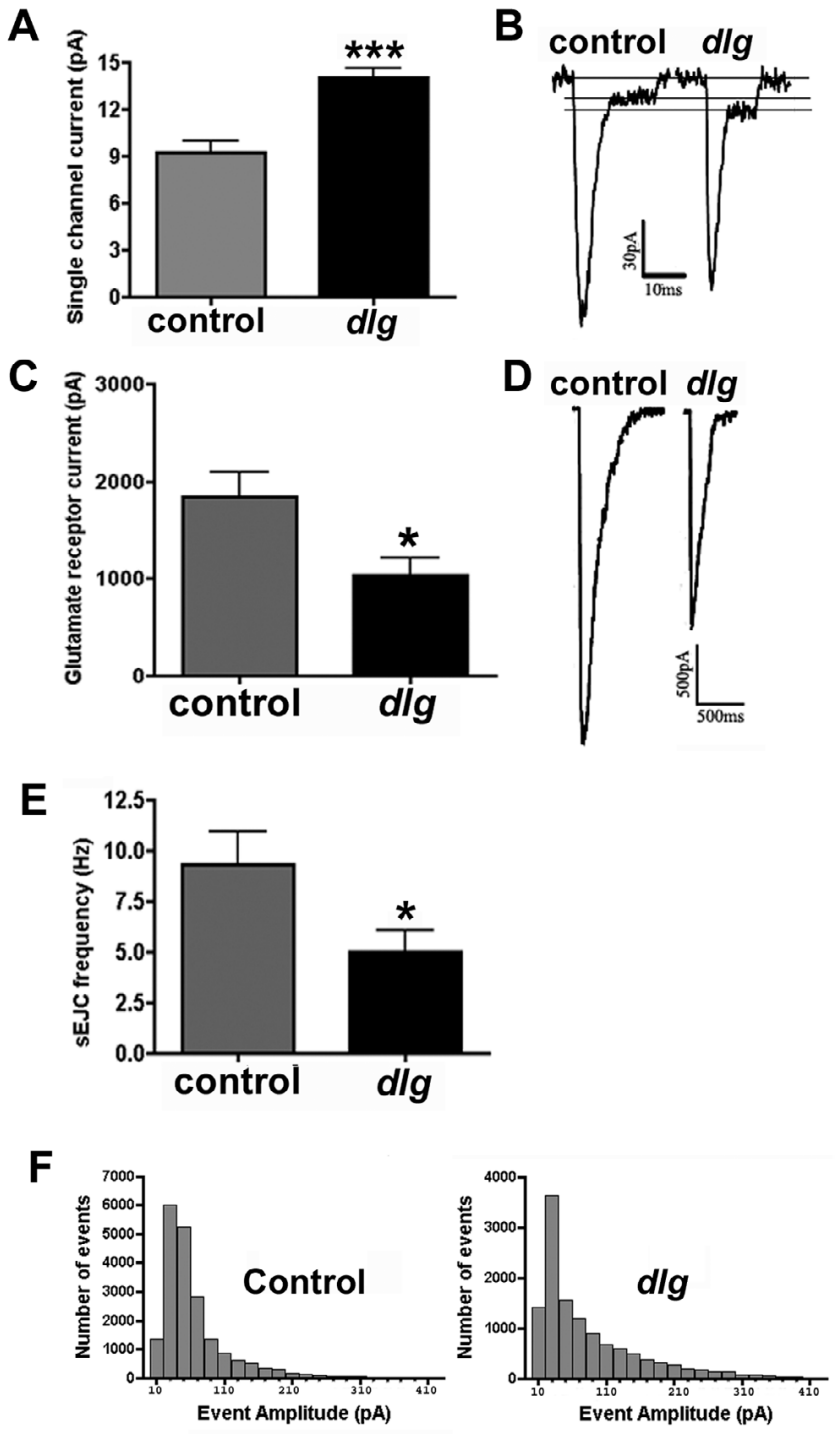
**Postsynaptic glutamate receptor current properties change in DLG mutants **A: Single glutamate receptor channel current amplitudes from synaptic glutamate receptors are significantly larger in *dlg *mutants, compared to controls. Single channel amplitudes were measured from channels displaying delayed closing during the falling phase of spontaneous synaptic currents; examples of sEJCs showing single channel currents are shown in B. C: Glutamate-gated currents, evoked using pressure ejection of 1 mM glutamate onto embryonic NMJs, are smaller in *dlg *mutants, compared to controls. Sample glutamate-gated currents are shown in D. E: The frequency of spontaneous excitatory junction currents (sEJCs) is reduced in *dlg *mutants, compared to controls. F: sEJC amplitudes are not significantly different in *dlg *mutants, compared to controls.

To confirm our immunocytochemical results, and explore the functional changes resulting from loss of GluRIIB-containing receptors, we used electrophysiology. First, we compared single glutamate receptor channel properties in control and *dlg *mutant embryonic muscle 6 (Fig. 3A–B). Because *Drosophila *glutamate receptor conductance is relatively large and embryonic muscle input resistance is relatively high, single glutamate receptor channel currents are visible during the falling phase of some spontaneous synaptic events (Fig. 3B). DiAntonio et al. [31] showed that extrasynaptic larval muscle glutamate receptors in GluRIIB null mutants have slightly larger single channel currents (8.8 pA and 9.2 pA at -60 mV, for wild-type larvae and GluRIIB null mutants, respectively). We saw a similar, but larger increase in synaptic receptor single channel amplitudes in *dlg *mutant embryos (Fig. 3A; control = 9.3 ± 0.7 pA at -60 mV, N = 17; *dlg *= 14.1 ± 0.06 pA at -60 mV, N = 42; P < 0.001).

DiAntonio et al. [31] also examined single channel kinetics in the absence of GluRIIA or GluRIIB. Although loss of GluRIIA resulted in a dramatic decrease in average open channel times, loss of GluRIIB did not result in any significant change in open channel duration, compared to wildtype. If *dlg *mutants selectively lose GluRIIB, but not GluRIIA, then there should correspondingly be no change in single glutamate receptor channel kinetics in *dlg *mutants. Consistent with this, we observed no change in average duration of single channel currents visible during the falling phase of spontaneous synaptic currents (control channel open time = 14.3 ± 1.5 ms, N = 17; *dlg *mutant channel open time= 12.1 ± 0.7 ms, N = 42; P = 0.13).

All evidence strongly suggests that there are two subtypes of ionotropic glutamate receptors at the *Drosophila *NMJ: 1) receptors that are made up of the subunits GluRIIA+IIC+IID+IIE, and 2) receptors that consist of GluRIIB+IIC+IID+IIE. Our immunocytochemical results (Fig. 2) suggest that in *dlg *mutants, GluRIIB-containing receptors are selectively lost without any compensatory increase in GluRIIA-containing receptors. If this is true, the total number of glutamate receptors measurable electrophysiologically should decrease. To test this prediction, we measured the amplitude of glutamate-gated currents triggered by pressure ejection of 1 mM glutamate onto postsynaptic muscles. Figure 3C–D shows that, glutamate-gated currents were significantly smaller in *dlg *mutants, compared to controls (control = 1842 ± 255 pA at -60 mV, N = 10; *dlg *= 1044 ± 173 pA at -60 mV, N = 10; P = 0.018). Dividing by the single channel current amplitudes allows us to calculate the number of individual receptors opened. Control currents represent the opening of approximately 198 (1842/9.3) receptors. Currents in *dlg *mutants represent the opening of approximately 74 (1044/14.1) receptors. Since pressure ejection of glutamate onto embryonic muscles activates extrasynaptic as well as synaptic receptors, this decrease in electrophysiologically detectable glutamate receptors also demonstrates that the loss of immunocytochemically visible receptors shown in Fig. 2 is not due to dispersal of GluRIIB-containing receptors away from postsynaptic sites.

Recent studies suggest that *Drosophila *NMJ glutamate receptors are specifically localized opposite active zones, and that GluRIIA-containing receptors and GluRIIB-containing receptors are segregated from each other [26,30,35]. In other words, it is thought that individual postsynaptic densities (PSDs) contain either GluRIIA or GluRIIB, but not both. If this is true, then loss of one receptor subtype should cause some active zones to be without apposing receptor fields, while other active zones have relatively normal receptor fields. Our electrophysiological results (Fig. 3C–D) show that 63% (±12%) of all receptors are missing in *dlg *mutants. If GluRIIA and GluRIIB are segregated into different PSDs, and GluRIIB-containing receptors are selectively lost in *dlg *mutants, then 63% (±12%) of the individual synapses (active zone-PSD pairs) should be silent in *dlg *mutants. This should show up as a decrease in spontaneous excitatory synaptic current (sEJC) frequency, without a corresponding decrease in sEJC amplitude. sEJC frequency drops to 54% (±14%) of normal in *dlg *mutants (Figure 3E; control = 9.3 ± 1.6 Hz, N = 13; *dlg *= 5.0 ± 1.0 pA, N = 13; P = 0.03). sEJC amplitude in *dlg *mutants, however, is not significantly different compared to controls (Fig. 3F; control = 79 ± 7 pA, N = 13; *dlg *= 69 ± 9 pA, N = 13; P = 0.41). These results are consistent with selective loss of GluRIIB-containing receptors in NMJs where individual postsynaptic densities are composed exclusively of receptors containing GluRIIA or GluRIIB.

### DLG mutants have fewer postsynaptic glutamate receptor clusters per presynaptic active zone

**Figure 4:**
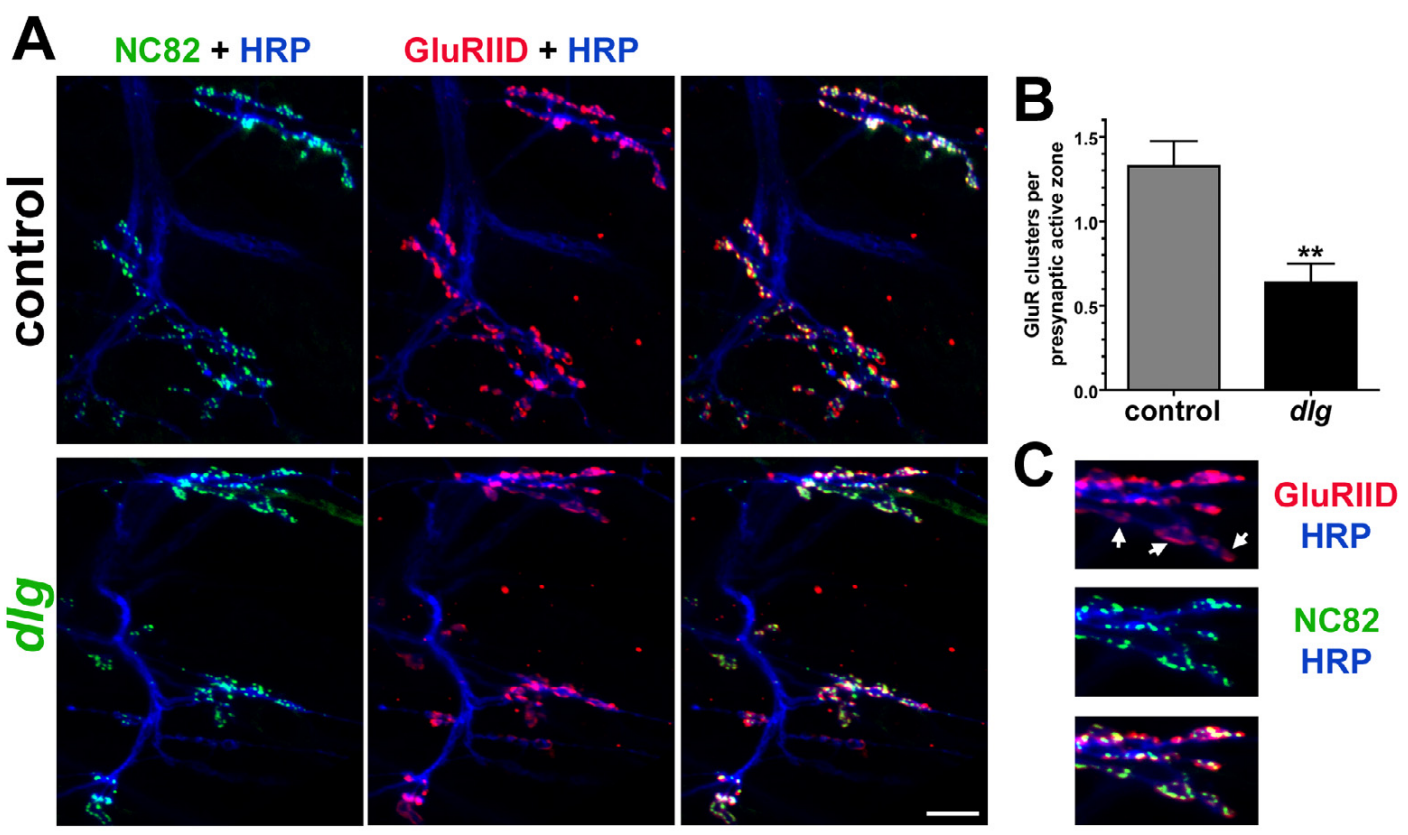
DLG mutants have fewer postsynaptic glutamate receptor clusters per presynaptic active zone.  A: Confocal projections of first instar NMJs visualized using three different antibodies: 1) the neuronal membrane marker anti-HRP (blue), 2) anti-GluRIID subunit antibodies which label all postsynaptic glutamate receptors (red), and 3) NC82 antibodies that label presynaptic active zones (green). Each panel shows NMJs on interior-most ventral longitudinal muscles in one hemisegment.  Scale bar = 10 um.  B: Number of postsynaptic glutamate receptor clusters relative to number of presynaptic active zones, showing that dlg mutants have fewer postsynaptic receptor clusters.  C: High magnification images from dlg mutant NMJs showing glutamate receptor dispersion (arrows).

If postsynaptic glutamate receptor clusters are composed of receptors containing either GluRIIA or GluRIIB, and GluRIIB-containing receptors are selectively lost in DLG mutants, then there should be fewer postsynaptic glutamate receptor clusters per presynaptic active zone in *dlg *mutants. We tested this by triple staining the first instar NMJs with anti-HRP antibodies to visualize the presynaptic nerve, NC82, an antibody that marks presynaptic active zones [38], and anti-GluRIID antibodies [26], which label all postsynaptic glutamate receptors. The results are shown in Figure 4. NC82 and GluRIID immunoreactivity appear as distinct puncta where motor neurons contact postsynaptic muscle (Fig. 4A). In control larvae, each NC82 punctum is associated with a GluRIID punctum. Not every GluR cluster is associated with NC82 or HRP immunoreactivity, however, consistent with the previously-described presence of extrasynaptic glutamate receptors [39-41]. Thus, the ratio of postsynaptic glutamate receptor clusters to presynaptic active zones is greater than one (Fig. 4B). Specifically, control larvae show an average glutamate receptor cluster to active zone ratio of 1.33. In dlg mutants, however, this ratio is reduced to approximately one-half normal (Fig. 4B; control = 1.326 ± 0.15 GluR clusters/active zone, N = 312 GluR clusters from 5 animals; *dlg *= 0.64 ± 0.11 GluR clusters/active zone, N = 231 GluR clusters from 5 animals; P = 0.006). These results are consistent with a model in which: 1) GluRIIB-containing receptors are clustered independently of GluRIIA-containing receptors, 2) GluRIIB-containing receptor clusters are selectively lost in *dlg *mutants, and 3) selective loss of GluRIIB-containing receptors causes some presynaptic active zones to no longer be associated with postsynaptic glutamate receptor clusters.

Interestingly, some receptor clusters opposite active zones in *dlg *mutants were visibly less distinct (Fig. 4C, arrows), suggesting that GluRIIB-containing receptors are not only lost, but some receptors are slightly mislocalized.

### Postsynaptic localization of GluRIIA, GluRIIB, and DLG requires contact by the presynaptic neuron

**Figure 5:**
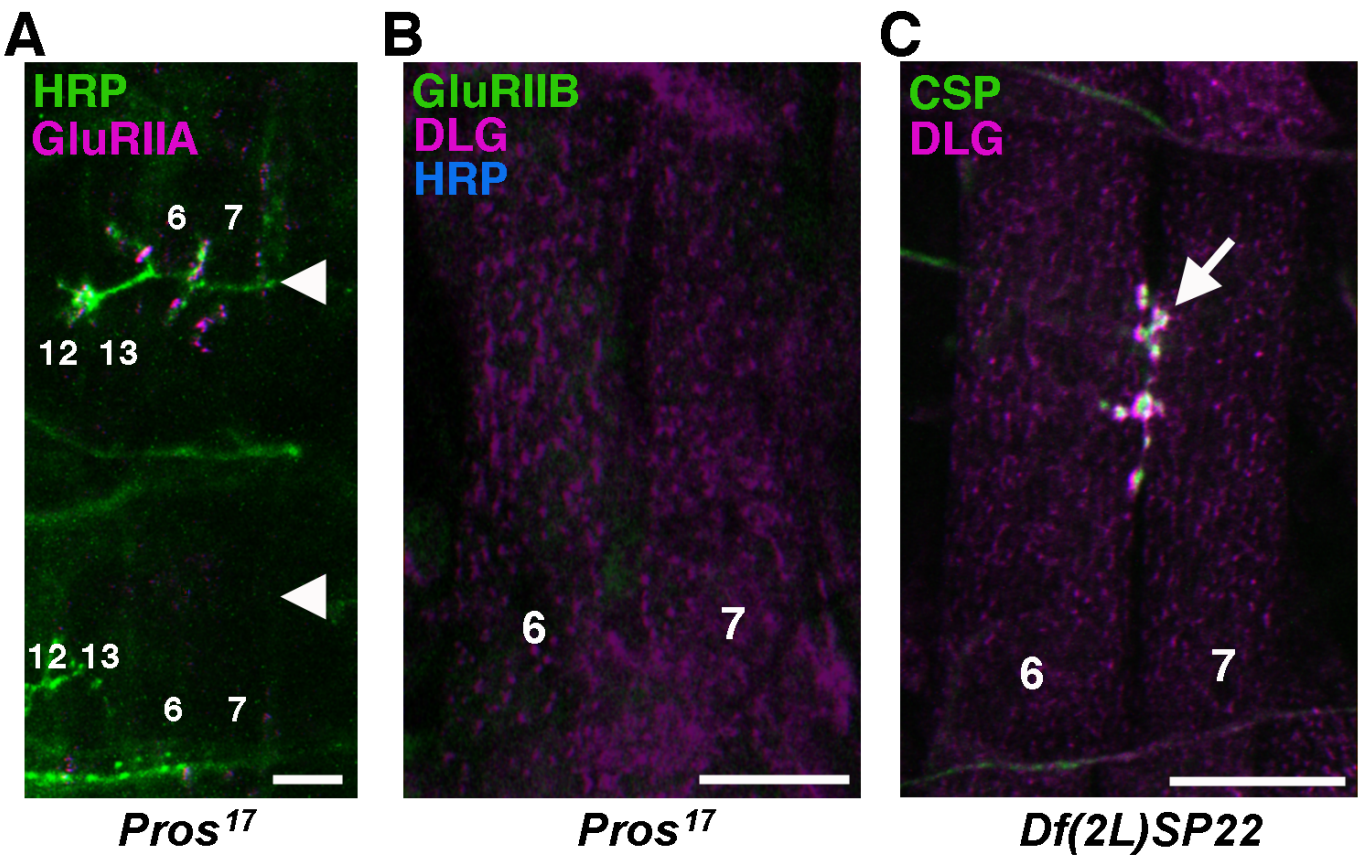
**Postsynaptic localization of GluRIIA, GluRIIB, and DLG requires contact by the presynaptic neuron, but localization of DLG does not depend on the presence of glutamate receptors **A: Confocal projections of late stage 17 embryonic NMJs visualized using antibodies to the neuronal membrane marker anti-HRP (green) and anti-GluRIIA subunit antibodies (magenta). This image shows NMJs on interior-most ventral longitudinal muscles in two neighbouring hemisegments in a *prospero *null mutant. The muscles in the upper hemisegment are normally innervated; the muscles in the lower hemisegment are not innervated. Major anatomical landmarks are labelled: In the upper hemisegment, intersegmental nerve branch b (ISNb) enters from the right (medial) and branches to form NMJs on muscles 7 & 6, 13, and 12. ISNb is absent in the uninnervated hemisegment. Note the lack of GluRIIA clusters in this hemisegment. B: Uninnervated muscles 6 & 7 in a *prospero *mutant embryo, stained using antibodies against GluRIIB, DLG, and the neuronal membrane marker HRP. Note the lack of GluRIIB clusters, and dispersal of DLG throughout the muscle membrane. C: Innervated muscles 6 & 7 in a GluR-less *Df(2L)SP22 *mutant embryo, stained using antibodies against the synaptic vesicle protein cysteine string protein (CSP, green)) and DLG (magenta). Note that DLG clusters properly at the synapse. Scale bars (A, B, C) = 15 μm.

Broadie & Bate [42] showed electrophysiologically that innervation triggers clustering and expression of functional glutamate receptors at the site of neuron-muscle contact. However, it has never been determined whether neuronal contact triggers localization of receptor protein, or local conversion of non-functional receptors to functional receptors. To answer this question, we repeated the critical experiments of Broadie & Bate but detected glutamate receptors immunocytochemically instead of electrophysiologically (Figures 5A–B).

In *prospero *null mutants, motor axon outgrowth is delayed and impaired, such that embryonic body wall muscles are variably innervated [42]. Fig. 5A shows two neighboring hemisegments in a late stage (24 h AEL) *prospero*^17 ^mutant embryo stained with anti-HRP to visualize motor axon terminals (green), and anti-GluRIIA antibodies (magenta). Ventral longitudinal muscles 12, 13, 6, and 7 (all unstained) are labelled in each hemisegment. The locations where the intersegmental nerve normally enter the ventral longitudinal muscle field in each hemisegment is marked with arrowheads. The muscles in the top hemisegment are innervated; ISNb (green) forms appropriate branches to each of the muscles, and GluRIIA clusters (magenta) are visible at the sites of innervation. As shown in Fig. 5A (top hemisegment, muscle 12), GluR clusters were typically visible even under growth cones, suggesting that receptors cluster at synaptic sites within minutes of nerve-muscle contact. In the neighboring uninnervated hemisegment (bottom of image), however, no GluRIIA clusters are visible. Thus, GluRIIA protein does not cluster in the absence of innervation. Similar results were obtained for GluRIIB (Fig. 5B). These results support the conclusion that postsynaptic glutamate receptors (containing either GluRIIA or GluRIIB) are clustered and upregulated only after contact by the presynaptic neuron. The signal between presynaptic neuron and postsynaptic muscle that triggers receptor clustering remains unknown.

Fig. 1 and previous studies [16-19,43] show that DLG (like GluRs) is largely restricted to the postsynaptic region. We used *prospero *mutants to determine whether DLG localization also depends on contact by the presynaptic neuron. Figure 5B shows muscles 6 and 7 in a non-innervated *prospero*^17 ^mutant hemisegment triple-stained for GluRIIB (green), DLG (magenta), and HRP (blue). As previously noted, glutamate receptor clusters are not visible in uninnervated muscles. Without innervation, DLG is also not localized; DLG remains dispersed throughout the muscle membrane in a pattern reminiscent of that seen when DLG is missing PDZ domains 1 and 2 [13]. In innervated muscles, DLG clusters appropriately at sites of muscle-nerve contact (Fig. 5C).

### Postsynaptic localization of DLG does not depend on the presence of glutamate receptors

Because glutamate receptor clustering requires neuron-muscle contact, and DLG clustering requires neuron-muscle contact, it is possible that DLG clustering requires glutamate receptors. We tested this by visualizing DLG in homozygous *Df(2L)SP22 *mutant embryos. *Df(2L)SP22 *mutants contain a deletion that removes the genes encoding GluRIIA and GluRIIB, resulting in complete loss of NMJ glutamate receptors [26,27,31]. Figure 5C shows an innervated hemisegment from a homozygous *Df(2L)SP22 *embryo, stained with antibodies to the presynaptic vesicle protein CSP (green) and DLG (magenta). The innervation-dependent clustering of DLG does not depend on glutamate receptors; DLG clusters opposite presynaptic boutons even in homozygous *Df(2L)SP22 *mutants (Fig. 5C). Note, however, that some extrasynaptic DLG remains. Extrasynaptic DLG remains prominent until approximately 48–60 h after hatching (mid second instar stage; data not shown).

## Discussion

We have tested for the first time whether DLG is required for formation and/or stability of postsynaptic glutamate receptors in *Drosophila*. Our results show that DLG is indeed required, but only for a subset of receptors, those that contain the subunit GluRIIB. The molecules required for similar assembly and/or stabilization of GluRIIA-containing receptors remain unidentified. The molecular mechanism by which DLG regulates GluRIIB stability remains unclear. There is currently no evidence for a direct interaction between DLG and any *Drosophila *glutamate receptor subunit. Genome-wide yeast two-hybrid assays failed to identify any interactions between DLG and any *Drosophila *glutamate receptor subunit [44]. Similar results were obtained in two other independent and otherwise successful yeast two-hybrid screens: One, using the C-termini of GluRIIA, GluRIIB, and GluRIIC as baits failed to identify any interaction with DLG (S Sigrist, personal communication). Another screen independently used the SAP97-like N-terminus, the PDZ1-2 domains and the GUK domain of DLG as baits, but failed to identify any glutamate receptor subunits (U Thomas, personal communication).

Despite the fact that DLG clearly regulates the number of GluRIIB-containing receptors in the *Drosophila *NMJ, we do not believe that DLG specifies the location of GluRIIB-containing glutamate receptors. First, glutamate receptors are clearly not localized based on DLG alone, because DLG is present extrasynaptically in uninnervated muscle (cf. Fig. 5), and abundant throughout the postsynaptic membrane (c.f. Fig. 1), but glutamate receptors are tightly localized to discrete puncta that are mostly (but not exclusively) found opposite presynaptic active zones [26,35]. Thus, DLG is not sufficient for glutamate receptor clustering or stability. Second, as described above, there is no evidence for a direct interaction between DLG and glutamate receptors. Our data are most compatible with a model wherein DLG participates in the stability of receptors (possibly by regulating the assembly of a 'stability-promoting complex'), but does not 'scaffold' receptors. This conclusion derives from the observations that immunoreactive GluRIIB-containing receptor clusters are largely absent in *dlg *mutants, and glutamate-gated currents are smaller in *dlg *mutants. If receptors were dispersed, clusters would disappear but glutamate-gated currents should remain normal. However, some declustering of receptors was observed in *dlg *mutants (Fig. 4C), and it is possible that unclustered receptors are endocytosed and/or rendered nonfunctional.

If DLG does not determine where receptors go, then something else must. We do not know the identity of this protein. We show that localization of postsynaptic DLG, like localization of postsynaptic glutamate receptors, depends on contact by the presynaptic neuron. We do not know the mechanism by which presynaptic contact triggers localization of either glutamate receptors or DLG. However, the identification of DLG as a target for this process should help identify the molecules involved in this critical initial trans-synaptic signal.

Our results are the first evidence that glutamate receptors in *Drosophila *can be differentially regulated based on subunit composition. Mammalian ionotropic glutamate receptors also undergo subunit-dependent assembly and trafficking, suggesting that receptor subunit-dependent interactions are a conserved method for 'tuning' postsynaptic properties. In the *Drosophila *NMJ, the most critical role for DLG may therefore be as part of the machinery for regulating subunit composition. One possible mechanism for this process could be as follows. In the *Drosophila *NMJ, active CamKII phosphorylates DLG [19]. Constitutively active CamKII increases extrasynaptic DLG and phenocopies *dlg *mutants [19]. Our results therefore predict that synaptic activity, via activation of CamKII, would decrease the number of GluRIIB-containing receptors and silence some synapses (active zone-PSD pairs). Sigrist et al. [45] showed that NMJ activity leads to enhanced translation and insertion of GluRIIA-containing receptors (they did not assay GluRIIB). Thus, the overall result of increased NMJ activity is probably replacement of GluRIIB-containing receptors with GluRIIA-containing receptors – a 'switch' in postsynaptic receptor subunit composition.

DiAntonio et al. [31] studied the effects of selectively expressing GluRIIA or GluRIIB transgenes in *Df(2L)SP22 *mutant *Drosophila*, where endogenous GluRIIA and GluRIIB were eliminated. The most dramatic changes in receptor properties resulted from overexpression of GluRIIA in the absence of GluRIIB: mEJP amplitudes increased several-fold, receptor channel open times increased, and sensitivity to the antagonist argiotoxin decreased. Thus, their results show that switching from 'B-type' receptors (e.g. those containing GluRIIB) to 'A-type' receptors (e.g. those containing GluRIIA) at the NMJ leads to changes in postsynaptic properties. Overexpression of GluRIIA increases presynaptic growth in larval NMJs [46], suggesting that postsynaptic subunit switching might also play a role in presynaptic development.

## Conclusions

Our results demonstrate that mutation of DLG causes loss of glutamate receptors containing GluRIIB, but not GluRIIA. We also show that, like glutamate receptors, DLG localization requires contact between pre and postsynaptic cells. DLG localization does not depend on the presence of glutamate receptors, since DLG is localized normally in the complete absence of postsynaptic glutamate receptors. Since glutamate receptor localization does not entirely depend on DLG, and DLG localization does not depend on glutamate receptors, we hypothesize that presynaptic nerve contact triggers localization of receptors and DLG in parallel, after which DLG promotes the stability of GluRIIB-containing receptors.

## Methods

### Genetics

'Control' genotypes were either Oregon R (OR) or non-homozygous mutant siblings of the appropriate genotype. No statistically significant difference in any measurement was observed between OR and any other control genotype used in this study. Homozygous mutant embryos were identified through the use of an appropriate balancer chromosome expressing GFP. *prospero[17] *mutants are nulls that were a gift from Dr Chris Doe, University of Oregon. *Df(2L)SP22 *mutants remove both GluRIIA and GluRIIB, as previously described [31]. *dlg[X1–2] *mutants [20] were gifts from Dr Vivian Budnik (University of Massachusetts Medical School).

### Immunocytochemistry

Embryos and larvae were dissected and stained for immunocytochemistry and electrophysiology as previously described [43]. When antibodies against any of the glutamate receptor subunits were used, preparations were fixed 30 min in Bouin's fixative. Otherwise, fixations were 30 min in 4% paraformaldehyde. Antibodies against GluRIIA (8B4D2, used at 1:100) [25] were produced from hybridoma cells and obtained from the University of Iowa Developmental Studies Hybridoma Bank (DSHB). Mouse NC82 antibodies were a gift from Erich Buchner and used at 1:100. Rabbit polyclonal GluRIIB antibodies [30] were used at 1:1000. Rabbit polyclonal anti-DLG antibodies [16] were used at 1:1000. Rabbit polyclonal GluRIID antibodies [26] were a gift from Stephan Sigrist and used at 1:500. All primary antibodies were visualized using fluorescently-conjugated (fluorescein, rhodamine, or CY-5) secondary antibodies (Jackson Immuno Labs, West Grove, PA) generated against the appropriate species (mouse or rabbit) and viewed using an Olympus FV-500 laser-scanning confocal microscope. Presynaptic terminals were visualized using fluorescently conjugated anti-HRP antibodies (Jackson Immuno Labs) directly conjugated to FITC, TRITC, or CY-5.

Receptor cluster sizes (Fig. 2C) were measured using an automated edge-finding/threshold-based macro run within NIH ImageJ software (v. 10.2 for OS X). Results using the automated procedure avoid experimenter bias and agree quantitatively with careful manual measurements [25]. The 3D reconstruction of the portion of a larval NMJ shown in Fig. 1B was generated using Amira 3.1 (Mercury Computer Systems, Chelmsford, MA).

The number of glutamate receptor clusters per active zone (Fig. 4) was quantified as follows: first instar larvae of the appropriate genotype were dissected and triple-stained with antibodies against NC82, which marks presynaptic active zones, GluRIID, which marks all postsynaptic glutamate receptor clusters, and HRP to visualize the NMJ. These preparations were subsequently imaged using confocal microscopy. Z-projections from each image (which contained several NMJs and dozens of clusters) were split into the separate color channels using ImageJ, and the number of clusters in each channel (NC82 or GluRIID) was counted using ImageJ's particle analysis function. The number of GluRIID clusters in each image was then divided by the number of NC82 clusters in each image to calculate ratios that were then compared using a Student's T-test (Fig. 4B).

### Electrophysiology

All electrophysiology (Fig. 3) was performed on ventral longitudinal muscle 6. whole-cell patch clamp measurements from embryonic muscles were performed as previously described [41,43]. Briefly, temporally and morphologically staged embryos were dechorionated in bleach, manually devitellinated and dissected, then treated with 1 mg/ml collagenase type IV (Sigma-Aldrich) for 60–90 s. Muscle 6 was whole-cell voltage clamped (-60 mV) in standard *Drosophila *embryonic saline using standard patch-clamp techniques. Data were acquired and subsequently analyzed using an Axopatch 1D amplifier and PClamp 9 (Axon Instruments, Union City CA).

### Statistics

Statistical significance in figures is represented as follows: *** = p < 0.001; ** = p < 0.01; * = p < 0.05. Unless otherwise specified (e.g. Fig. 2C, 3F), all statistical comparisons were made using unpaired T-tests, or (in the case of distributions) Kolmogorov-Smirnov tests. All error bars represent S.E.M.

## Authors' contributions

All immunocytochemistry and microscopy was performed and analyzed by DEF. All electrophysiology was performed and analyzed by KC. The manuscript was written by DEF with input from KC; both authors reviewed and approved the final manuscript.
